# Refracture after plate removal of midshaft clavicle fractures after bone union—incidence, risk factors, management and outcomes

**DOI:** 10.1186/s12891-023-06391-0

**Published:** 2023-04-19

**Authors:** Yurun Zhu, Jianping Hu, Taicheng Zhan, Kunpeng Zhu, Chunlin Zhang

**Affiliations:** Department of Orthopedic Surgery, Shanghai Tenth People’s Hospital, Tongji University School of Medicine, #301 Yanchang Middle Road, Shanghai, 200072 P.R. China

## Abstract

**Introduction:**

There is a great debate on the routine use of open reduction and internal fixation (ORIF) for midshaft clavicle fractures, and one concern is the adverse events after ORIF, such as implant removal after bone union. In this retrospective study, we assessed the incidence, risk factors, management and outcomes of refracture after plate removal of midshaft clavicle fractures after bone union.

**Materials and methods:**

Three hundred fifty-two patients diagnosed with acute midshaft clavicle fractures who had complete medical records from primary fractures to refracture were recruited. Details of imaging materials and clinical characteristics were carefully reviewed and analysed.

**Results:**

The incidence rate of refracture was 6.5% (23/352), and the average interval from implant removal to refracture was 25.6 days. Multivariate analysis showed that the risk factors were Robinson type-2B2 and fair/poor reduction. Females were 2.4 times more likely to have refracture, although it was not significant in multivariate analysis (p = 0.134). Postmenopausal females with a short interval (≤ 12 months) from primary surgery to implant removal had a significant risk for refracture. Tobacco use and alcohol use during bone healing were potential risk factors for male patients, although they were not significant in multivariate analysis. Ten patients received reoperation with or without bone graft, and they had a higher rate of bone union than 13 patients who refused reoperation.

**Conclusion:**

The incidence of refracture following implant removal after bone union is underestimated, and severe comminute fractures and unsatisfactory reduction during primary surgery are risk factors. Implant removal for postmenopausal female patients is not recommended due to a high rate of refracture.

**Supplementary Information:**

The online version contains supplementary material available at 10.1186/s12891-023-06391-0.

## Introduction

In recent decades, open reduction and internal fixation (ORIF) has become more accepted for displaced or comminute midshaft clavicle fractures [1, 2]. However, great concern remains regarding the complications following ORIF, such as infection and skin irritation, which may lead to additional operations, such as implant removal. As the most common complication after ORIF, implant removal of clavicle fixation after bone union is not routinely recommended and performed. However, for clavicle fracture, the number of patients who request implant removal after bone union is huge due to skin irritation, cosmetic reasons or psychological discomfort of retained [3, 4].

Implant removal after bone union may result in subsequent complications such as infection, haematoma and persistent pain. One of the complications is refracture, which is a rare event and is mostly reported in fractures located in the forearms and in other long bones, such as the femur shaft [5, 6]. Although the refracture rate is 2–5%, it is a serious complication, which in most cases makes a reoperation necessary [6, 7]. For midshaft clavicle fractures, there is minimal literature reporting on refracture, although there is a huge demand for implant removal. In this retrospective study, we aimed to assess the incidence, risk factors, management and outcomes of refracture after implant removal of midshaft clavicle fractures after bone union.

## Patients and methods

Patients who had undergone plate removal of midshaft clavicle fractures after ORIF from July 2013 to June 2019 were enrolled in this retrospective study. The beginning date was set because we could not retrieve the imaging materials of patients registered before July 2013 in the electronic medical records system. The inclusion criteria were as follows: (1) patients who were diagnosed with acute midshaft clavicle fractures based on clinical and imaging findings; (2) patients who received ORIF surgery using plates and screws and underwent subsequent plate removal after bone union in our hospital; (3) patients who were skeletally mature at the time of the primary surgery; and (4) patients who had complete imaging materials and medical records from primary fracture to refracture. The exclusion criteria were as follows: (1) patients who underwent removal of plate and screws before fracture union due to complications, including infection, nonunion or periprosthetic fracture that required refixation; and (2) patients with incomplete medical records and/or with a shorter follow-up. The study was approved by the IRB in our hospital, and 352 patients were included in the final analysis.

Data on age, gender, body mass index (BMI), status of menopause, diabetes mellitus (DM), side of the procedure, concurrent injury, alcohol use, tobacco use, and interval from primary surgery to implant removal were collected. Imaging materials (mainly X-ray) were carefully reviewed by the senior doctor (Dr. ZCl) to record the Robinson classification, reduction quality, bone healing and refractures. Fracture union was defined as follows: (1) resolution of the fracture line on radiographs (X ray or CT), (2) painless weight-bearing, and (3) no or minimal tenderness to palpation over the fracture site. All patients in the current study met the criteria of fracture union, and implant removal was not performed without senior doctors’ approval. Delayed union was defined as a prolonged time from definitive surgery to fracture union greater than 12 weeks. Fair/poor reduction was defined as reduction with either presentation as follows: (1) displacement > = 2 mm between fracture ends or fracture fragments, (2) shortening > = 10 mm of whole clavicle length, (3) angulation, rotation or lateral displacement, and (4) segmental loss of fracture fragment. Secondary fracture at the same site or at screw holes of the clavicle 3 months after implant removal was considered to be related to previous clavicle fracture and ORIF and was defined as refracture.

During implant removal surgery, the fracture site was examined via the naked eye and confirmed by intraoperative fluoroscopy after the plate and screws were removed. All fracture sites were stable, and solid union of the fracture was obtained. Patients were allowed to make full range motions without restrictions after removal of plate and screws but were not allowed to do weight-bearing activities during the first month. Thereafter, these patients were allowed to perform weight-bearing activities as tolerated after senior doctors’ approval.

The demographic characteristics of these patients are summarized in Table 1. A 2-tailed Student’s t test for continuous variables and a chi-square test for categorical variables were used to compare the groups. Risk factors (predictors) were checked by univariate analyses, and odds ratios (ORs) with 95% CIs were calculated. Then, a multivariate logistic regression model including all the potential predictors was performed. Predictors that had a P value of the log likelihood ratio test greater than 0.10 were excluded (one by one) until all the remaining predictors had P values less than 0.10. Significance was set at P < 0.05, and SPSS Version 20.0 (SPSS Inc., Chicago, IL) was used for all statistical analyses.

**Table 1:** Patients’ characteristics (BMI: body mass index).

	Refractures(n = 23)	No refracture(n = 329)	p-value
Female n(%)	11(47.8%)	95(28.9%)	0.055
Male n(%)	12(52.2%)	234(71.1%)	
Age			
< 50	8(5.2%)	146(94.8%)	0.370
>=50	15(7.6%)	183(92.4%)	
BMI			
< 18.5	0(0%)	9(100%)	0.795
>=18.5&<24	13(7.0%)	172(93%)	
>=24&<28	9(6.8%)	125(93.2%)	
>=28	1(3.8%)	25(96.2%)	
Tobacco use, n (%)	3(13.0%)	14(4.3%)	0.091
Alcohol use, n (%)	2(8.7%)	7(2.1%)	0.111
Diabetes mellitus, n (%)	3(13.0%)	32(9.7%)	0.607
Left-sided procedure, n (%)	9(39.1%)	161(48.9%)	0.363
Concurrent injuries, n (%)	3(13.0%)	51(15.5%)	0.752
Robinson classification			< 0.001
2A	2(2.9%)	66(97.1%)	
2B1	4(1.9%)	210(98.1%)	
2B2	17(24.3%)	53(75.7%)	
Lock plate use,n(%)	19(82.6%)	280(85.1%)	0.762
Interfragmentary screws used n(%)	12(52.2%)	148(45.0%)	0.523
Fair/poor reduction n(%)	16(69.6%)	45(13.7%)	< 0.001
Delayed union or malunion n(%)	9(39.1%)	25(7.6%)	< 0.001
Mean interval between fixation and removal (weeks)	62.48	67.65	0.268
Interval stratification n (%)			0.620
<=12 months	4(11.8%)	30(88.2%)	
> 12&<=18 months	13(6.3%)	194(93.7%)	
> 18 &<=24 months	4(5.4%)	70(94.6%)	
> 24 months	2(5.4%)	35(94.6%)	

## Results

We reviewed 352 patients: 246 were men, 106 were women, the average age was 48 ± 12.9 years, the mean body mass index (BMI) was 23.7 ± 3.0 kg/m^2^, and the mean interval from primary surgery to plate removal was 67.3 ± 21.6 weeks. Ultimately, refracture occurred in 23 patients, and the incidence rate was 6.5% (23/352). Twenty-two patients felt sudden pain and disability during activities of daily life without full weight-bearing (e.g., put on clothes, reach out for things, or take a bath), and only one patient fell at home. Twenty-one patients had a fracture at the previous fracture site, and 2 had a fracture at a screw hole. The mean interval between plate removal and refracture was 25.6 days (range 2–87 days).

As shown in Table 1, the refracture group tended to have more female patients, although though the difference was not significant (p = 0.055). In the refracture group, more patients reported tobacco use or alcohol use during bone healing, and the rates of Robinson classification type-2B2, fair/poor reduction and delayed union or malunion were also remarkably high. There was no significant difference between patients with refracture and those without refracture regarding age, BMI, diabetes mellitus, concurrent injuries, left side, use of lock plate, use of interfragmentary screws and interval between fixation and plate removal.

**Table 2:** Risk Factors for refractures (Univariate and multivariate logistic regression models, those variables with p-value < 0.10 were not presented)

	Univariate analysis OR (95% CI)	P value	Multivariate analysis OR (95% CI)	P value
Female vs. Male	2.258(0.963–5.294)	0.061	2.377(0.766–7.372)	0.134
Tobacco use vs. none	3.375(0.896–12.714)	0.072	2.959(0.114–76.743)	0.154
Alcohol use vs. none	4.381(0.856–22.411)	0.076	2.334(0.053-101.947)	0.660
Robinson classification		< 0.001		< 0.001
2 A	Ref.		Ref.	
2B1	0.629(0.113–3.509)		0.494(0.083–2.942)	
2B2	10.485(2.340-47.872)		7.901(1.571–39.740)	
Fair/poor reduction vs. good reduction	14.425(5.623–37.011)	< 0.001	15.783(5.337–46.675)	< 0.001
Delayed union or malunion vs. common procedure	7.817(3.080–19.840)	< 0.001	1.231(0.308–4.192)	0.769

In univariate analysis, female sex, tobacco use, alcohol use, Robinson classification type-2B2, fair/poor reduction and delayed union or malunion were potential risk factors. However, in multivariate analysis, only Robinson classification type-2B2 and fair/poor reduction remained as risk factors. Patients with fractures classified as type 2B2 were 7.9 times more at risk for refracture than those classified as type 2 A, and fair/poor reduction was associated with 15.8 times the risk for refracture (Table 2).

**Table 3:** Subgroup analysis of the 106 female patients (BMI, body mass index).

	Refractures(n = 11)	No refracture(n = 95)	p-value
Menopause n(%)	9(81.8%)	37(38.9%)	0.009
Non-menopause n(%)	2(18.2%)	58(61.1%)	
Age			
< 50	3(7.5%)	37(92.5%)	0.450
>=50	8(12.1%)	58(97.9%)	
BMI			
< 18.5	0(0%)	6(100%)	0.792
>=18.5&<24	8(11.6%)	61(88.4%)	
>=24&<28	3(10.3%)	26(89.7%)	
>=28	0(0%)	2(100%)	
Diabetes mellitus, n (%)	2(18.2%)	8(8.4%)	0.294
Left-sided procedure, n (%)	3(27.3%)	44(46.3%)	0.363
Concurrent injuries, n (%)	0(0%)	12(12.6%)	0.211
Robinson classification			< 0.001
2A	2(9.5%)	19(90.5%)	
2B1	1(1.6%)	63(98.4%)	
2B2	8(38.1%)	13(61.9%)	
Lock plate use,n(%)	9(81.8%)	80(84.2%)	0.838
Interfragmentary screws used n(%)	5(45.5%)	34(35.8%)	0.528
Fair/poor reduction n(%)	8(72.7%)	14(14.7%)	< 0.001
Delayed union or malunion n(%)	4(36.4%)	7(7.4%)	0.015
Meaninterval between fixation andremoval (weeks)	62.09	68.62	0.268
Interval stratification n (%)			0.052
<=12 months	3(27.3%)	8(72.7%)	
> 12 months	8(8.4%)	87(91.6%)	

Female patients were more likely to experience refracture, although the difference was not significant. Among 106 female patients, 11 patients had refracture. In the refracture group, patients were more likely to have gone through menopause, have delayed union or malunion and have a short interval (≤ 12 months) between fixation and removal (Table 3). Multivariate analysis showed that postmenopause and a short interval (≤ 12 months) from primary surgery to implant removal were risk factors in addition to the Robinson classification type-2B2 and fair/poor reduction. Tobacco use and alcohol use during bone healing were potential risk factors for male patients, although they were not significant in multivariate analysis. Robinson classification type-2B2 and fair/poor reduction remained risk factors for male patients in multivariate analysis (Table 4).

**Table 4:** Risk Factors of refractures regarding to gender. (Univariate and multivariate logistic regression models, those variables with p-value < 0.10 were not presented)

	Univariate analysis OR (95% CI)	P value	Multivariate analysis OR (95% CI)	P value
**Female**				
Menopause vs. non-menopause	7.054(1.443–34.477)	0.016	11.546(1.068-124.867)	0.044
Interval stratification < = 12 months vs. > 12 months	4.078(0.899–18.491)	0.068	105.887(4.641-2415.698)	0.003
Robinson classification		0.002		0.028
2 A/2B1	Ref.		Ref.	
2B2	16.821(3.945–71.720)		15.259(1.334-174.509)	
Fair/poor reduction n(%)	15.429(3.644–65.320)	< 0.001	23.070(2.051-259.516)	0.011
Delayed union or malunion vs. common union	7.184(1.686–30.615)	0.008	1.300 (0.127–13.286)	0.825
**Male**				
Tobacco use (TU) vs. No TU	5.238(1.274–21.539)	0.022	3.056(0.108–86.763)	0.513
Alcohol use (AU) vs. No AU	6.486(1.192–35.300)	0.031	2.443(0.050–119.300)	0.653
Robinson classification		< 0.001		< 0.001
2 A/2B1	Ref.		Ref.	
2B2	14.550(3.771–56.139)		16.252(3.397–77.740)	
Fair/poor reduction vs. Good reduction	28.909(7.284–114.730)	< 0.001	20.537(4.310–97.850)	< 0.001
Delayed union or malunion vs. common union	8.571(2.470-29.747)	0.001	0.522(0.066–4.105)	0.537

**Fig. 1:**
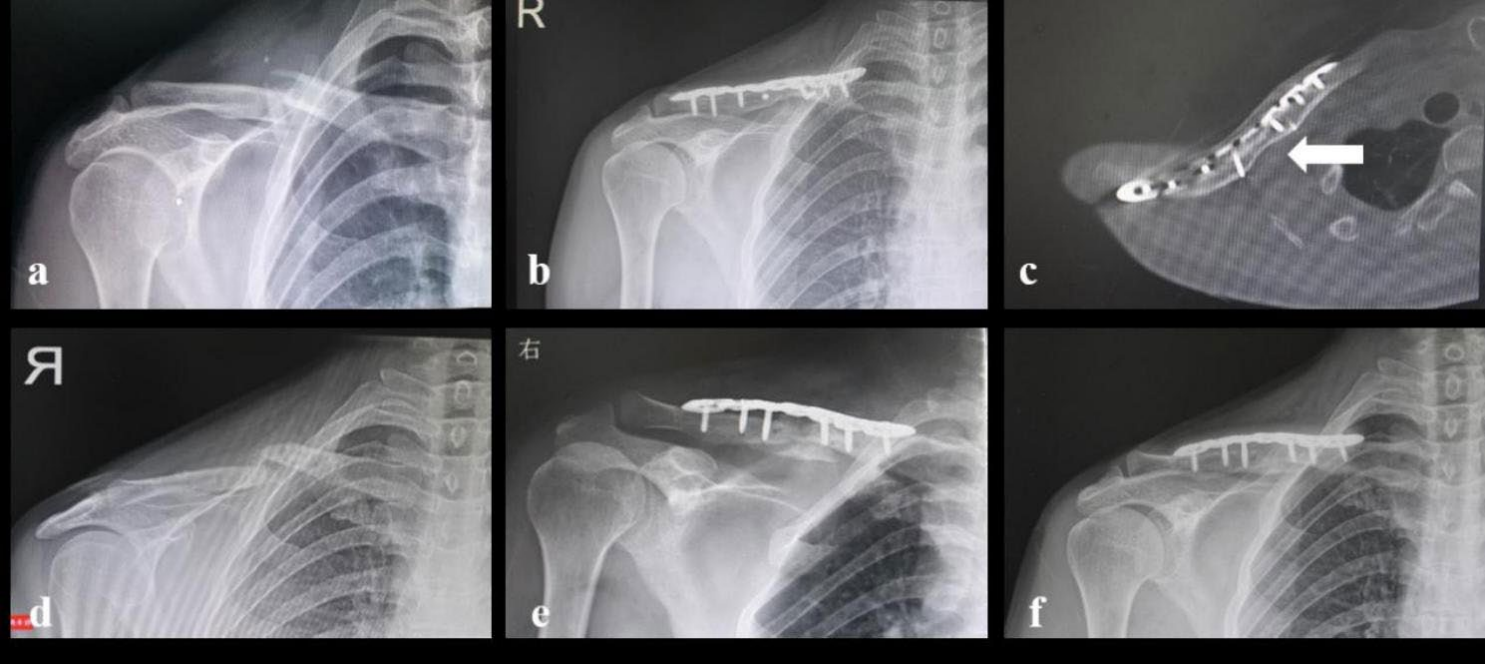
A female patient (54 years old) had a midshaft clavicle fracture (Robinson 2B2) after a fall to ground (a). She received ORIF and achieved bone union (b) and insisted on implant removal though CT scan showed that the bone quality at previous fracture site was not strong as that of surroundings (c, white arrow, 108 weeks after primary operation). She had implant removed and unfortunately refractures occurred at previous fracture site 30 days after implant removal (d). Reoperation with autogenous iliac bone graft (black arrow) was applied (e) and she achieved bone union 3 months later (f)

Ten of 23 patients who had a refracture received a second operation of ORIF; of them, 3 patients received ORIF combined with autologous iliac bone graft or cancellous allograft (Fig. 1). All ten patients had fractures united at the final follow-up, and 4 had plates removed; no refracture occurred again. Thirteen patients refused a second operation and were treated with figure-eight bandages and/or arm slings. Of them, three patients did not achieve solid bone union 6 months after refracture and requested an operation, and 6 patients experienced delayed union (Table S1).

## Discussion

In this retrospective study, we reviewed the imaging materials and clinical data of 352 patients who had complete medical records from primary fracture to refracture. We found that the incidence of refracture following implant removal after bone union was 6.5%, an underestimated rate that was thought to be rare (1–5%) in previous [8, 9]. Coincidentally, according to a recent retrospective study on refracture of midshaft clavicle fracture, the incidence was 7.2%, which is similar to our [10].

Such a refracture rate cannot be ignored when we considering plates as an operation strategy for midshaft clavicle fractures. The main disadvantages of open reduction and plate fixation are complications such as infection and skin irritation, and more than one in four patients need to have their implants [4]. Refracture after implant removal, as an additional adverse event, may raise the question on whether open reduction and plate fixation is a cost-effective strategy. There is a growing demand for implant removal although routine ORIF for midshaft clavicle fracture is not recommended, and the pros and cons should be carefully weighed before making decisions [2].

Such a refracture rate also indicates the potential adverse impact of implant removal. Implant removal is not recommended as a routine procedure for fractures after bone union. Ample evidence suggests that implant removal does not relieve the symptoms related to implants, and complications related to implant removal are not [11, 12]. Indeed, after implant removal of the clavicle, screw holes in clavicles may change the biomechanical effects on stiffness and load to [13]. Meanwhile, decreasing the strength of the clavicle after implant removal may induce [14, 15]. Thus, taking the cost‒benefit ratio into account, it is important to identify the risk factors for refracture before making the decision on implant removal after bone union.

In the current study, the Robinson classification type-2B2 was a risk factor for refracture. According to the Robison classification, there is no residual contact between the major fragments and variable degrees of shortening clinically and radiologically in type-2B. Type-2B fractures are further divided into simple or wedge comminuted fractures (type-2B1) and isolated segmental or segmentally comminuted fractures (type-2B2) [16]. Robinson type-2B2, which indicates the severity of comminuted fractures and less attachment of soft tissue, is a risk factor for adverse bone healing outcomes [16–18]. Moreover, the severity of comminuted fractures increases the difficulty of reduction. Although interfragmentary lag screws or cerclage wires (metal wires or absorbable suture lines, seldom used) have been applied to fix fracture fragments in the current study, some patients still cannot heal or accept reduction, and the rate of fair/poor reduction is 17.3% (61/352). Unaccepted reductions, such as shortening ( > = 10 mm) [19] and displacement or loss of segmental [20], are the highest radiographic risk factors for adverse bone healing quality.

Comminuted fractures and unaccepted reduction may result in poor united bone quality, which may lead to refracture. Stress shielding underneath metal fracture plates may interfere with normal bone healing and potentially cause considerable loss of bone [21], while comminuted fractures and unaccepted reduction worsen the situation. Ample evidence suggests that regional bone quality significantly correlates with the incidence of [22, 23]. Regional bone quality (bone mineral density, cortical thickness) has many effects on bone responses to pressure and shear forces until failure, i.e., fracture [24, 25]. Although we cannot obtain data on bone mineral density or cortical thickness of the previous fracture site because CT scans are not routinely performed to assess bone union before implant removal, the fact that most refracture occurred at the previous fracture site in the current study indicates the loss of bone quality.

In addition to comminuted fractures and unaccepted reduction, postmenopause may also contribute to the loss of bone quality in female patients. As we found in this study, postmenopause is a potential risk factor for refracture. In the current study, the mean age of female patients was 49 years old, which is a common menopausal age in China. Postmenopausal females suffer from oestrogen deficiency and are more likely to have low bone mineral density or postmenopausal osteoporosis. Several studies have found that oestrogen deficiency impairs both the normalization of mechanical properties and the accretion of minerals by the fracture [26]. According to a recent large-size study, fracture repair in postmenopausal women is flawed, and there is a significant decline in bone mineral density in the cortex and a reduction in bone strength and stiffness two years after [27]. Additionally, a short interval between primary surgery and implant removal was significantly associated with refracture in female patients in the current study, which indicates that female patients need more time to recover and return to having normal bone quality after bone fracture. For male patients, tobacco use and alcohol use during bone healing were potential risk factors in this study. Smoking and drinking after fracture are thought to be associated with adverse events such as infection, delayed union and [28]. Cigarette addiction and heavy drinking are related to decreased bone mineral density and can suppress intracortical bone [29, 30].

In the current study, management of refractures after removal mainly depended on the severity of fractures. These patients who had a refracture did not experience a high-energy injury but were mostly affected during daily life tasks, and the Robinson classification was mainly type 2 A or 2B1. Conservative treatment with figure-eight bandages and/or arm slings was applied to 13 patients, and 10 patients achieved bone union six months later, while 3 patients requested surgery without signs of bone union. In the group who received reoperation initially, 3 patients used bone grafts to accelerate bone union, and all patients achieved bone union. According to our results, initial reoperation tended to bring a higher rate of bone union. However, the recommendation of reoperation with or without bone graft is still not sound, and it needs to be confirmed via large-series and well-controlled studies.

There are several limitations in this study. First, this is a retrospective observation study with a variable number of factors included, which can lead to selection bias. However, our study consisted of a large number of patients, which can minimize bias. Second, operations for all fractures and implant removal were not performed by one surgeon, and the difference in experience between each surgeon may lead to bias in the operation results and subsequent outcomes. Fortunately, all decisions and operations were supervised by senior doctors. Third, CT scan or bone mineral density examination was not routinely performed before implant removal. Bone quality after bone union cannot be confirmed, which can weaken our hypothesis on the potential mechanism of risk factors for refracture.

## Conclusion

The incidence of refracture following implant removal after bone union is underestimated, and severe comminute fractures and unsatisfactory reduction during primary surgery are risk factors. Implant removal for postmenopausal female patients is not recommended due to the high rate of refracture.

## Electronic supplementary material

Below is the link to the electronic supplementary material.

Supplementary Material 1

## Data Availability

The data that support the findings of this study are available from the corresponding author upon reasonable request.

## Abbreviations

ORIF: Open reduction and internal fixation
BMI: Body mass index
DM: Diabetes mellitus
ORs: Odds ratios
