# Supplementary material for: Refracture after plate removal of midshaft clavicle fractures after bone union—incidence, risk factors, management and outcomes
Source: BMC Musculoskelet Disord. 2023 Apr 19;24:308. doi: 10.1186/s12891-023-06391-0 (PMC10114427; doi:10.1186/s12891-023-06391-0)
Supplement: Supplementary file 1 — Supplementary Material 1 [file 12891_2023_6391_MOESM1_ESM.docx]

Table S1 Clinical characteristics of 23 patients who had refractures and subsequent management and outcomes.

| Patients | Gender/age (years) | DM, Tobacco use or alcohol useor postmenopausal | Robinson classification | Reduction quality | Delayed union or malunion | Removal interval from ORIF (weeks) | Refracture interval from implant removal(days) | Management | Outcomes |
| --- | --- | --- | --- | --- | --- | --- | --- | --- | --- |
| 1 LWL | F/31 | none | 2B2 | Fair/poor | none | 44 | 9 | Conservative treatment | Nonuion |
| 2 XYQ | F/21 | none | 2A | Good | none | 48 | 7 | Reoperation without bone graft | Bone union |
| 3 CLH | F/63 | Postmenopausal | 2A | Good | none | 41 | 4 | Conservative treatment | Delayed union |
| 4 NPF | F/55 | Postmenopausal | 2B2 | Poor | Delayed union | 61 | 8 | Reoperation with bone graft | Bone union |
| 5 ZZY | F/55 | DM, Postmenopausal | 2B2 | Poor | none | 58 | 8 | Conservative treatment | Delayed union |
| 6 MXH | F/52 | Postmenopausal | 2B2 | Fair | none | 64 | 25 | Reoperation with bone graft | Bone union |
| 7 ZQF | F/56 | DM,Postmenopausal | 2B2 | Poor | none | 54 | 28 | Conservative treatment | Bone union |
| 8 HJ | F/49 | Postmenopausal | 2B1 | Good | Delayed union | 56 | 43 | Conservative treatment | Delayed union |
| 9 LJ | F/58 | Postmenopausal | 2B2 | Poor | Delayed union | 73 | 32 | Conservative treatment | Nonunion |
| 10 YW | F/55 | Postmenopausal | 2B2 | Fair | none | 76 | 33 | Conservative treatment | Nonunion |
| 11 ZYP | F/54 | Postmenopausal | 2B2 | Fair | Delayed union | 108 | 30 | Reoperation with bone graft | Bone union |
| 12 QDB | M/57 | Smoking/Drinking | 2B2 | Fair | Delayed union | 48 | 2 | Conservative treatment | Bone union |
| 13 GLM | M/45 | Smoking | 2B2 | Poor | Delayed union | 51 | 32 | Conservative treatment | Bone union |
| 14HSW | M/51 | none | 2B2 | Fair | none | 63 | 87 | Reoperation without bone graft | Bone union |
| 15 XHB | M/29 | none | 2B2 | Fair | Delayed union | 58 | 56 | Conservative treatment | Bone union |
| 16 LFS | M/63 | none | 2B2 | Good | no | 54 | 2 | Reoperation without bone graft | Bone union |
| 17 XHS | M/32 | none | 2B2 | Good | none | 62 | 54 | Conservative treatment | Bone union |
| 18 XJQ | M/57 | none | 2B1 | Poor | none | 51 | 2 | Conservative treatment | Delayed union |
| 19 ZHB | M/57 | Smoking and Drinking | 2B1 | Good | none | 52 | 60 | Reoperation without bone graft | Bone union |
| 20 ZYH | M/46 | DM | 2B1 | Good | none | 51 | 38 | Reoperation without bone graft | Bone union |
| 21 ZWQ | M/50 | none | 2B2 | Poor | Delayed union | 73 | 8 | Reoperation without bone graft | Bone union |
| 22 XNX | M/52 | none | 2B2 | Fair | Delayed union | 83 | 11 | Conservative treatment | Delayed union |
| 23 JSH | M/47 | none | 2B2 | Fair | Delayed union | 108 | 9 | Conservative treatment | Delayed union |
